# A Liquid-Metal-Based Dielectrophoretic Microdroplet Generator

**DOI:** 10.3390/mi10110769

**Published:** 2019-11-11

**Authors:** Ronghang Wang, Lunjia Zhang, Meng Gao, Qifu Wang, Zhongshan Deng, Lin Gui

**Affiliations:** 1 Key Laboratory of Cryogenics, Technical Institute of Physics and Chemistry, Chinese Academy of Sciences, Beijing 10019, China; wangronghang14@mails.ucas.ac.cn (R.W.); zhanglunjia14@mails.ucas.ac.cn (L.Z.); mgao@mail.ipc.ac.cn (M.G.); wangqifu16@mails.ucas.ac.cn (Q.W.); zsdeng@mail.ipc.ac.cn (Z.D.); 2 School of Future Technology, University of Chinese Academy of Sciences, Beijing 100039, China

**Keywords:** liquid-metal electrodes, dielectrophoretic (DEP) force, droplet generation

## Abstract

This paper proposes a novel microdroplet generator based on the dielectrophoretic (DEP) force. Unlike the conventional continuous microfluidic droplet generator, this droplet generator is more like “invisible electric scissors”. It can cut the droplet off from the fluid matrix and modify droplets’ length precisely by controlling the electrodes’ length and position. These electrodes are made of liquid metal by injection. By applying a certain voltage on the liquid-metal electrodes, the electrodes generate an uneven electric field inside the main microfluidic channel. Then, the uneven electric field generates DEP force inside the fluid. The DEP force shears off part from the main matrix, in order to generate droplets. To reveal the mechanism, numerical simulations were performed to analyze the DEP force. A detailed experimental parametric study was also performed. Unlike the traditional droplet generators, the main separating force of this work is DEP force only, which can produce one droplet at a time in a more precise way.

## 1. Introduction

Droplet-based microfluidics have been extensively applied in multiple fields, including cosmetics, medicine [1,2,3], chemistry [4,5], and biological detections [6]. Usually, droplets are manipulated by several methods, to implement droplet splitting [7], merging [8,9,10], and migration [10]. Particularly, the droplet can load certain quantities of samples, including proteins [1], cells [11], and even bacteria solutions. Using droplets as reaction vessels facilitates the measuring rate [1], enhances reaction rate [2], and even eradicates the contamination [6]. Because of its remarkable superiority, there are copious amounts of research on how to generate droplets in an efficient and source-saving way.

There are active or passive methods to generate droplets. Concretely, an active method utilizes an external force to cut one or several droplets from bulk fluid, and the cutting time is always decided by manipulators. While in a passive method, the droplets are continuously generated when the generating system is set. Droplets generated via a passive method are always identical. There are two typical passive generators: the T-junction [12,13,14] generator and the flow-focusing [15,16,17,18,19,20,21] generator. They both use two immiscible fluids to generate droplets and control the size of droplets by modulating the flow rates or the pressure of two phases [22]. Although the passive control possesses multiple superiorities, like the high monodispersity [15,16,17,18,19,20,21] of droplets, the high-throughput, and the marvelous automation, it still has a fatal drawback, low operation flexibility, resulting in the impossibility of producing diversities of droplets. It is also hard to operate robustly [23] and control the droplet-production time [24], since the long-lived and chaotic oscillations exist in the devices, causing the flow to vary uncontrollably and making it difficult to reach a steady state [25]. In contrast, the active methods can generate droplets and control droplet size promptly and precisely. The utilized external forces always stem from the application of these technologies, including thermodynamics [26,27,28,29,30], mechanics [31,32,33,34,35,36], magnetism [14,37], and electricity [24,38,39,40,41,42,43,44,45,46]. 

Electricity is one of the most powerful, convenient, and promising methods to generate droplets precisely and instantly [24]. Electrical control comprises direct current (DC) control and alternative current (AC) control. Initially, electricity was introduced to the mechanical control to facilitate the procedure of obtaining ultra-fine droplets [46]. The electric field force in a flow-focusing generator made it possible to produce charged droplets as small as femtoliters and recombine, separate, and sort these droplets quickly [24]. The AC also facilitated the stable emulsification process at high voltage, producing tiny droplets (less than one microliter) through the formation of Taylor cone [47]. Furthermore, the utilization of AC electric field directly at the flow-focusing junction promoted the precise modification of droplet sizes in the emulsification process [48], without any environmental influence.

AC control contains two forms, the low-frequency (30–300 kHz) control and high-frequency (3–30 MHz) control. Initially, AC control was applied in the exploration of forming droplets and the observation of the relaxation oscillation phenomenon in a low-frequency electric field [36,49]. High-frequency control was also used to generate droplets, the size of which can be adjusted by modulating the frequency and root-mean-square amplitude of the AC voltage [11,36]. Although AC could generate droplets precisely, it still has some transparent drawbacks.

The droplet-producing process and results based on electricity are restricted by several factors. The side effects of the external environment still existed until the sealed channels occurred in the electrowetting experiments [50]. Electrolysis caused by espousing electrode to the electrolyte limited the ceiling capability of electrowetting (less than several hundred millivolts [51]) until the electrowetting on the insulator-coated electrode (EICE) [52] appeared. The integration of EICE and the flow-focusing configuration facilitated the droplet transferring [53], which modulated the droplet size and produced frequency by adjusting the applied voltage and the pulses [54], merged droplets with high efficiency up to 98% [55], and guaranteed the detailed on-demand droplet generation [55]. The application of finer orifice in the design also enhanced the precise modulation in the droplet dimension [55,56,57], and the results showed that droplet size increased with the increase of applied voltage amplitude in the conical-spraying regime [57]. The accumulation of surfactant also reduced the droplet size and enabled it to be around 1 to 2 microns in the tip-streaming regime [58]. Later, the implementation of Norland Optical Adhesive (NOA) material promoted the small aspect ratio of channels [59] and lifted the low stiffness restriction for the device. Eventually, the non-contact electrode [48,60] fabricated by injection ceased the fouling of the solution and electrode in the device [61] and reduced the droplet-formation time to milliseconds [62].

The electricity (like electrowetting) only acted in a supporting role until the dielectrophoresis (DEP) appeared in the droplet generators. Initially, T.B.Jones et al. [63] proposed a smart DEP generator to form finite droplets (~60 nanolitres [64]) after the voltage is removed. Then, RaviPrakash et al. [65] utilized this theory and demonstrated an extensively rapid and well-controlled microactuator of aqueous samples, with dispensers that varied from nanolitre to picolitre. Even though these DEP droplet choppers above can form droplets instantly with the assistance of specific structures, they cannot work well without the unique structures.

Herein, we presented a universal and robust active microdroplet generator based on the dielectrophoresis theory. The device had a T-junction in the flow channel where the two phases met and a controllable interface was formed. Different from the devices using AC electric field as an assistant tool in the flow-focusing field [48], this droplet generator directly utilized DC electric field to introduce DEP forces as the main separating force to generate size-controllable droplets. It is easy to obtain droplets with different sizes by modulating the electrode length and distribution, changing the value of voltages applied on the certain electrode, or modulating the interface of two phases. Therefore, these “scissors” can provide droplets with a range of sizes and be applied to medicine [1,2,3] and chemistry [4,5] fields. Simulations were demonstrated to understand how the DEP force sheared liquid into drops and analyze the droplet-generation process clearly. Finally, the exhaustive experimental parametric study was implemented. The proposed device may have potential applications in drug screening, single-cell extraction, pharmaceutical synthesis, biochemistry detection, etc.

## 2. Experiments and Methods

### 2.1. Dielectrophoresis Droplet Generator

The active T-junction droplet generators were fabricated by the standard soft lithography [66]. The transparent mask with the design was utilized to create an SU-8 2050 (MicroChem Corp., Westborough, MA, USA) mold for microchannels. After being vacuumed for over 2 hours, in order to remove the bubbles, Polydimethylsiloxane (PDMS), the Sylgard 184 silicone elastomer (a mixture of a base and a curing agent at a 10:1 ratio per weight, Dow Corning Corp.) was poured onto the SU-8 2050 master mold. Then, the PDMS was heated on the hot plate at 65 °C, for around 2.5 hours before it was peeled off from the mold. The fluid and electrodes’ channels had the same height (50 μm) since they were also patterned simultaneously. This PDMS slab with channels was then bonded to another empty PDMS slab after air plasma treatment (plasma cleaner, YZD08-2C, Tangshan Yanzhao Technology).

**Figure 1:**
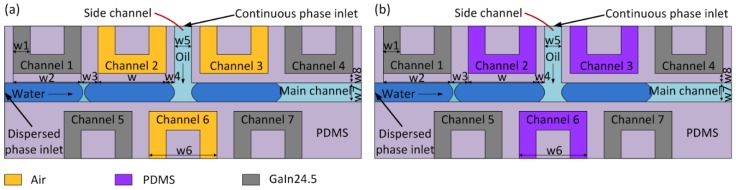
Structure of the dielectrophoresis droplet generator. (**a**) Droplet generator of scheme 1; (**b**) droplet generator of scheme 2. The only difference between scheme 1 and scheme 2 was the filling material of non-electrode Channel 2/3/6, with air in scheme 1 and PDMS in scheme 2.

There are two distinct schemes to generate droplets. Figure 1 showed that scheme 1 and scheme 2 had the same design size, while the material-filled-in-Channels 2/3/6 were different. The material-filled-in-Channels 2/3/6 in scheme were PDMS, while that in scheme 2 was air. To form electrodes, Channels 1/4/5/7 were assembled by Ga75.5In24.5 (75.5 wt.% Ga, 24.5 wt.% In, melting point: 15.7 °C; Shanxi Zhaofeng Gallium Co., Ltd., Yangquan, Shanxi, China) via injection with syringe pumps (LSP10-1B, Longer Precision Pump Co, Ltd., Baoding, China). The width w1 and w2 were 70 and 470 µm, separately. Furthermore, the gaps between Channels 1/2/3/4/5/6/7 (w3) were 80 µm. The gaps between Channels 2/3 and the side channel (w4) were 30 µm. Meanwhile, the width of the side channel (w5) and the main channel (w7) were both 60 µm. Moreover, the gap between Channels 1/2/3/4/5/6/7 and the main fluidic channel (w8) were 30 µm. Furthermore, the width of Channels 2/3 (w) and that of Channel 6 (w6) were both 470 µm.

### 2.2. Droplet Generation System

**Figure 2:**
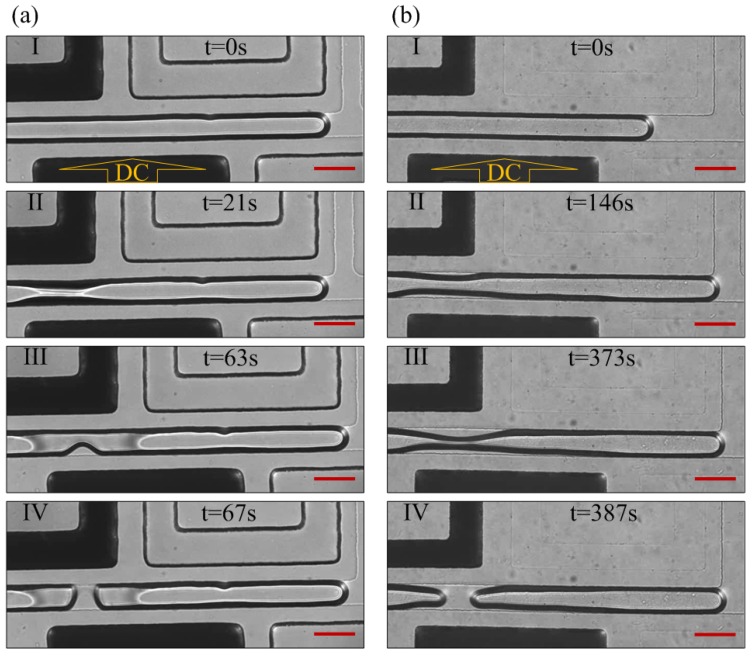
The droplet generation process under 2600 V voltage. (**a**) Droplet generation process in scheme 1; (**b**) droplet generation process in scheme 2. Voltage was applied on the electrode marked as “DC” (Channel 5). The scale bars are 100 µm.

Deionized water and silicon oil were pumped into the channels at constant pressure, using a microflow static pressure system (MFCSTM-EZ, Fluigent, Villejuif, France). Because this droplet-generation system is not used for generating a lot of droplets continuously at a time, no surfactant is necessary to added into the system to prevent uncontrollable coalescence [47]. First, the whole system was filled with silicon oil to form a very thin oil-film on the inner surface of the microchannels. This ultra-thin oil-film will help the silicon oil flow easily along the wall to “cut” the water. Then, the water was pumped into the main channel from the dispersed phase inlet as shown in Figure 1. The silicon oil was pumped into the side channel from the continuous phase inlet. After that, the flow pressure of the water (*P*_water_) and that of the silicon oil (*P*_silicon oil_) were adjusted to around 50 mbar and 40 mbar, respectively, and a stable interface between water and silicon oil was formed near the junction of main channel and side channel. The uneven electric field was induced by applying voltage on just one electrode with a high voltage power supply (HVS448 6000D, Lab Smith, Inc., Livermore, CA, USA). The voltage utilized ranged from 2600 to 3000 V. The voltage was only applied on the Channel 5 (marked as “DC” in Figure 2), leaving others grounded. All the videos and pictures were recorded by a fluorescence microscope (Axio Observer Z1, Carl Zeiss, Oberkochen, Germany). 

## 3. Results and Discussion

### 3.1. Droplet Generation

**Figure 3:**
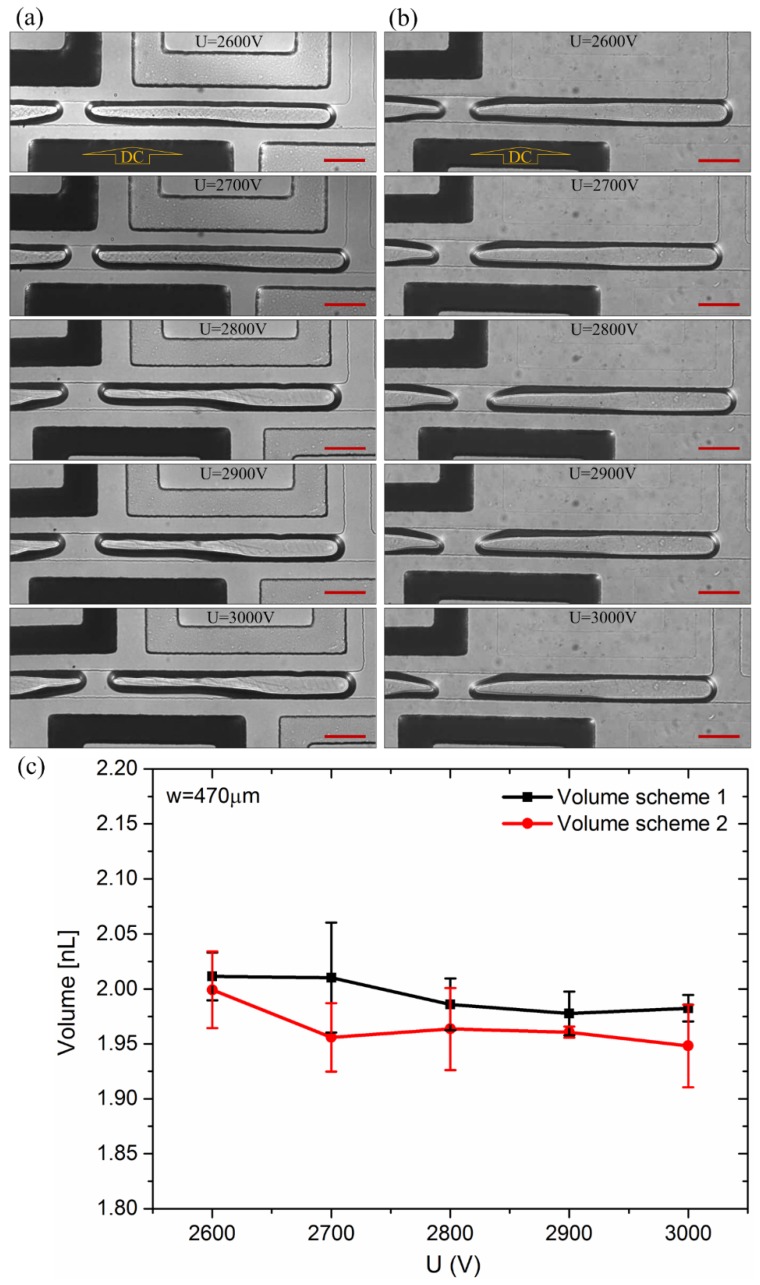
Droplets formed in different voltage in schemes. (**a**) The droplets formed in scheme 1; (**b**) the droplets formed in scheme 2; (**c**) the volume of the droplet in schemes 1 and 2 under the voltage from 2600 to 3000 V. The relative error was less than 3%. Voltage was applied on the electrode marked as “DC”. The scale bars are 100 µm.

Figure 2 demonstrated that the voltage (2600 V) applied to electrode marked as “DC” induced the meniscus at nearly the same location both in schemes 1 and 2, after the interface was formed. The liquid column was torn into two and generated a droplet around the meniscus place in both schemes. The length of the droplet in the two schemes was almost the same (around 660 µm). Notably, a similar phenomenon also appeared when the voltage changed to 2700, 2800, 2900, and 3000 V. All the experiments under different occasions were performed three times. As shown Figure 3, the pinch-off point was a little closer to the upstream at low voltage, while that shifted further toward downstream at higher voltage. The volume of droplets indicated small differences which could be ignored in scheme 1 and scheme 2 (Figure 3), with the relative error of less than 3%.

The water column break-up and droplet generation process contains four main steps (Figure 2). (I) The deionized water was pumped into the main channel and stopped with its tip staying at the T-junction by carefully controlling the pressure of the two phases. (II) The voltage was applied and a necking area began to show up. (III) The neck shrinked continuously as the silicon oil accumulated in the vicinity. (IV) The neck broke and formed a new droplet at its right side. The next step was to remove voltage and reduce *P*_silicon oil_ to transfer the droplet downstream. A new cycle would not begin until the interface stabilized at the T-junction by carefully controlling *P*_water_ and *P*_silicon oil_ again. The potential field acted like “invisible scissors”, cutting the water into droplets as desired.

The reason for the droplet generation can be explained as follows. The voltage applied to the electrode induced an uneven electric field, which could move the polarized water molecules via the electric field forces. Since the intensity and direction of the electric field force applied to the molecules were different, the molecules would migrate to different places with diverse velocities [67,68]. The dielectrophoretic (DEP) force acting on a dipole could follow Equation (1):

(1) $$\vec{F}=\vec{m}\times \nabla \vec{E}$$

where $\vec{m}$ represents the dipole moment of the particle, and $\vec{E}$ represents the electric field strength [67]. Supposing polarized water molecules as particles $\vec{m}$ could be described as Equation (2):

(2) m→=4πεmRe[CM(ω)]r3E→

where $\varepsilon_{m}$ represents the dielectric permittivity of silicon oil; *r* represents the radius of a particle; and Re[CM(ω)] represents the Clausius–Mossotti factor. Then, the DEP force could be simplified as Equation (3):

(3) F→=2πεmr3Re[CM(ω)]∇E→2

As for the water in oil with the voltage frequencies ranging from zero to several MHz, Re[CM(ω)] equals to 1 [69]. Our experiments were under this condition as DC voltage was applied to the electrode. There was a presumption that the DEP force acted on the deionized water could induce the orientated migration of the water molecules, which induced the orientated migration of the liquid. Thus, the DEP force triggered the droplet generation. As the silicon oil accumulated around the snip-off point (the trigger point), the water column also bore the shear stress from the silicon oil. Eventually, the water column broke into two, and the droplet was generated. Since the droplet generation triggering process depended on the value of DEP force, droplet generation occurred in scheme 1 and scheme 2 only when the DEP force applied on the trigger point was large enough. Similarly, the break-up point or the length of droplet also varied with the voltage applied on the electrode, because the change of electric field led to the change of DEP force. 

### 3.2. Simulation and Parametric Study

**Figure 4:**
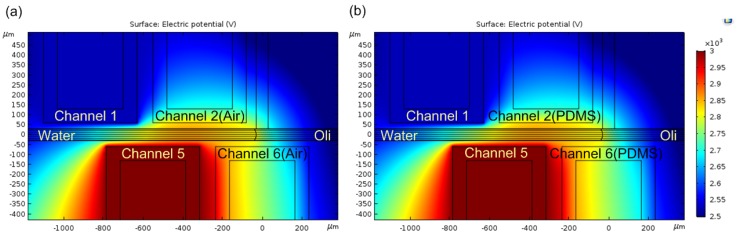
Electric potential distribution of scheme 1 and scheme 2. (**a**) Electric potential distribution of scheme 1; (**b**) electric potential distribution of scheme 2. The difference was the filling material of non-electrode Channel 2/6, with air in scheme 1 and PDMS in scheme 2. Three thousand volts was applied on Channel 5.

To gain a better understanding of the droplet-formation principle, numerical simulations were implemented, using COMSOL. The geometry of the channels was the same as the chips in experiments. The *x* axis is set along the main channel, and the *y* axis is set along the side channel. Models neglected the affect of Channel 3, Channel 4, and Channel 7 since they rarely influenced theoretic and experimental results. Here, we suppose voltage applied on Channel 5 was 3000 V since the voltage value change (ranging from 2600 to 3000 V) had a moderate effect on the break-up point. As is apparent from Figure 4, there was no distinct difference between the electrical potential of scheme 1 and scheme, but it showed that there was an obvious sharp decrease between the left and the right of the the intersection of the pair of electrodes.

**Figure 5:**
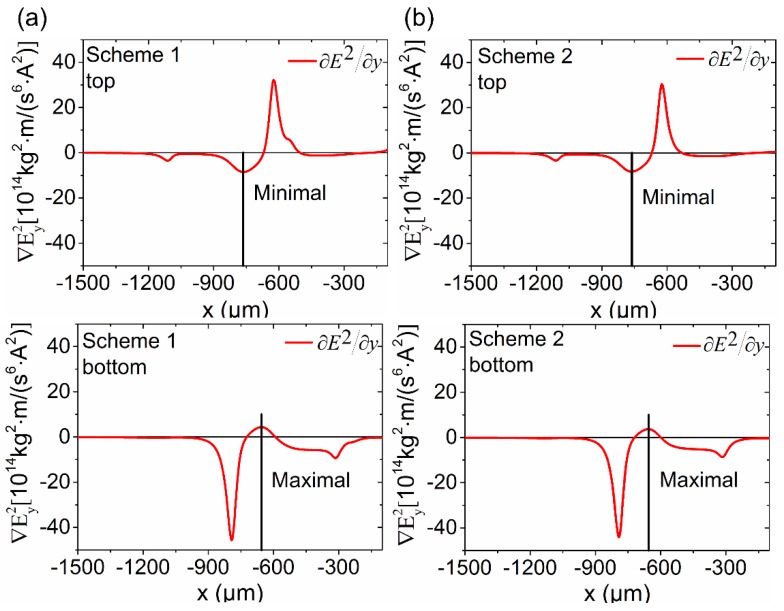
Distribution of $\nabla E_{y}^{2}$ near the top wall and bottom wall (at the distance of 2 µm from the top and bottom wall). (**a**) The distribution in scheme 1; (**b**) the distribution in scheme 2. The minimal and maximal represented the extreme value, and the droplet generation would be activated at these points, where the DEP force was the greatest off the wall.

According to Equation (3), DEP force is directly proportional to $\nabla {\vec{E}}^{2}$. Thus, $\nabla E_{y}^{2}$ represents the DEP force applied on the water on the *y*-direction. To explain the droplet generation, as shown in Figure 5, distribution of $\nabla E_{y}^{2}$ near the top wall and bottom wall were simulated (at the distance of 2 µm from the top and bottom wall). According to the numerical results, the distribution of $\nabla E_{y}^{2}$ seemed almost the same in schemes 1 and 2, which agree with the experimental results shown in Figure 3. The snip-off point should occur at the minimal value of $\nabla E_{y}^{2}$ (negtive) at the top wall and the maximal value of $\nabla E_{y}^{2}$ (positive)at the bottom wall, where the largest DEP force occurs and try to drive water off the wall. The DEP force on the y-direction leads to the necking.

**Figure 6:**
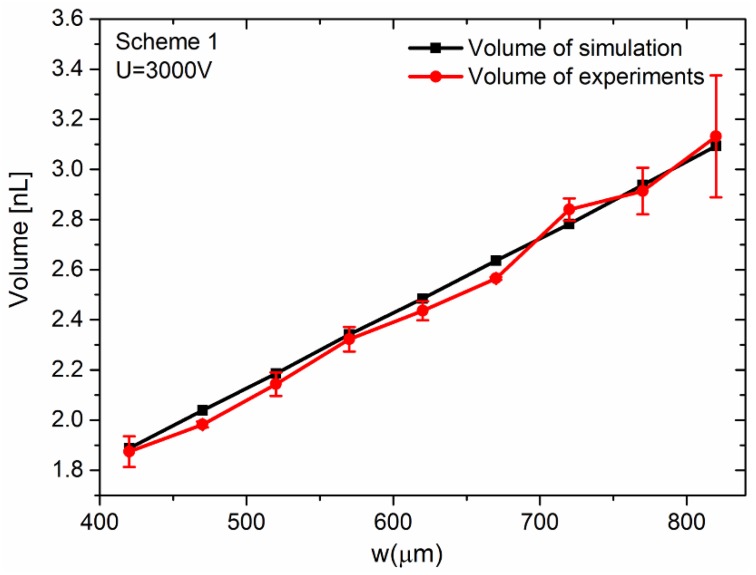
The volume of the droplet in simulation and experiments under the voltage of 3000 V in scheme 1. The w = 420, 470, 520, 570, 620, 670, 720, 770, and 820 µm.

Near the top wall, the minimal value in scheme 1 and scheme 2 bottomed at nearly –8.53 × 10^14^ kg^2^·m/(s^6^·A^2^) at −764 µm and −8.27 × 10^14^ kg^2^·m/(s^6^·A^2^) at −762 µm, respectively. Near the bottom, the maximal value in schemes 1 and 2 peaked at around 4.36 × 10^14^ kg^2^·m/(s^6^·A^2^) near –644 µm and 3.69 × 10^14^ kg^2^·m/(s^6^·A^2^) around –647 µm, separately. It meant that the DEP force could trigger droplet generation around these points as the magnitude of the extrusion force there was almost 10 times of that in other places. Generally, the droplet generation only occurred when the DEP force was strong enough. Clear from Figure 3, the place where droplets were generated was near the intersection of the pair of electrodes. It was not hard to see that it was near the midpoint of the top break-up point and the bottom break-up point in simulation results. Thus, we took the midpoint as the point where droplet generation was activated. As shown in Figure 6, the simulation results were highly consistent with the experiments. 

The droplet formation can be explained as a result of the interaction between the DEP force acting on the water and the shear force from silicon oil. Due to the extrusion force from the top and bottom, the neck of the water column shrank and triggered the accumulation of silicon oil there. With the continuous accumulation of oil, the water column bore the shear force from the oil, resulting in a slender neck. Eventually, the neck was snipped off when it could not bear the DEP force and the shear stress from silicon oil, generating a droplet with a certain length. It is not difficult to generate a droplet with a certain length when the interface between water and oil is fixed or defined by the “T-junction”.

Thus, the pair of electrified electrodes worked like a pair of “invisible” scissors to cut the droplet off from the bulk. Using these “scissors”, the volume of droplet can be well controlled by designing the distribution of the electrodes. As shown in Figure 6, the volume of the droplet varies with *w* in Scheme 1. The w ranged from 420 to 820 µm, with an interval of 50 µm, and the volume of the droplet got larger with it. We did three sets of experiments for each value of w. Figure 6 indicated that the numerical simulation matched the corresponding experimental results well. As shown in Figure 6, the volume of the droplet is highly linear with w. The maximal relative error of the experiments was less than 8%. The cause of error between the numerical results and experimental results came mainly from the slight flunctuation of the interface between oil and water at the T-junction, and the inaccuracies of the channel parameters during the fabrication process. Furthermore, the slight flunctuation of the interface might be the factor bringing the droplet volume change under the same voltage, as the break-up point was fixed. The difference of the gap w8 between experiments and simulations also made the shift of the electric field, leading to the position change of the break-up point.

**Figure 7:**
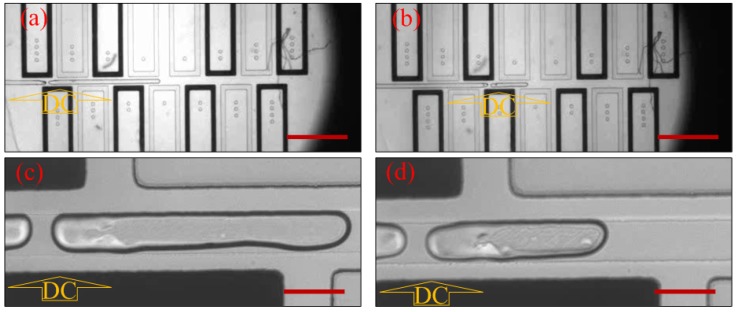
The droplet generation under 3000 V voltage. The scale bar in (**a**) and (**b**) is 1000 µm; the scale bar in (**c**) and (**d**) is 100 µm. Voltage was applied on the electrode marked as “DC”.

On the other hand, besides designing different value of w to control the droplet size, the droplet volume can also be controlled in a more “dynamic” way. As shown in Figure 7, a series of electrodes can be fabricated in a column beside the main channel in advance. By choosing which pair of electrodes to be electrified, the length and volume of droplet can be easily controlled. As shown in Figure 7, with the fixed interface at the T-junction, when the first pair of electrodes on the left (Figure 6a,c) was electrified, a longer droplet was generated, while a shorter droplet was generated (Figure 6b,d) with the second pair of electrodes being electrified. Thus, the same droplet generator could actively generate droplets with different size by just switching different pairs of electrodes. The applied voltage was 3000 V.

## 4. Conclusions

In this work, we proposed a novel droplet generator based on liquid-metal electrodes and explored the principle of droplet formation in an uneven electric field. Obviously, this device could generate droplets with a certain length and volume when the interface between two phases was stabilized at the T-junction. The electrified liquid metal electrodes worked like invisible “scissors” to cut the droplet off from the bulk. To reveal the mechanism of the “cutting”, a model was built based on the DEP force, and this model could successfully predict the position of the snip-off point of the droplet. By designing different sized electrodes, the droplet size can be controlled from chip to chip. The droplet size can also be easily controlled by selecting which pair of electrodes is to be electrified in a series of electrodes on the same chip. This DEP droplet generator could be useful in extensive applications, such as medicine, biologic tests, and cosmetics.
